# Community health workers supporting diverse family caregivers of persons with dementia: Preliminary qualitative results from a randomized home-based study

**DOI:** 10.1177/14713012241302367

**Published:** 2024-11-18

**Authors:** Jung-Ah Lee, Julie Kim, Julie Rousseau, Eilleen Sabino-Laughlin, Eunae Ju, Eunbee Angela Kim, Amir Rahmani, Lisa Gibbs, Adeline Nyamathi

**Affiliations:** Sue & Bill Gross School of Nursing, 8788 University of California, Irvine, USA; Department of Sociology, 8788 University of California, Irvine, CA, USA; Sue & Bill Gross School of Nursing, 8788 University of California, Irvine, USA; Division of Geriatric Medicine and Gerontology, Department of Family Medicine, School of Medicine, 8788 University of California, Irvine, USA; Sue & Bill Gross School of Nursing, 8788 University of California, Irvine, USA; Division of Geriatric Medicine and Gerontology, Department of Family Medicine, School of Medicine, 8788 University of California, Irvine, USA; Sue & Bill Gross School of Nursing, 8788 University of California, Irvine, USA

**Keywords:** caregiver, community health worker, culturally sensitive, dementia, linguistically appropriate

## Abstract

**Background and Objectives:**

Culturally diverse informal caregivers of community-dwelling persons with dementia face challenges in accessing dementia care resources due to language barriers and cultural stigmas surrounding dementia. This study presents the perceived intervention experiences of a home-based approach which considers the cultural and linguistic needs of diverse family caregivers in dementia care. The intervention model includes home visits by trained bilingual, non-licensed community health workers (CHWs) whose cultural histories and understandings reflect that of the caregivers. The purpose of the present study was to understand family caregivers’ experience in caregiving and their feedback on the intervention, which includes caregiver support through education and skill development.

**Methods:**

The present study thematically analyzed qualitative data from exit interviews with caregivers who participated in a CHW-led, 12-week home visit-based intervention program.

**Results:**

Among 57 caregivers (mean age = 63.5, SD = 14.3) who participated in the 3-month home-visit intervention and completed the exit interviews, 33% were Korean Americans, 28% Vietnamese Americans, 21% non-Hispanic Whites, and 17.5% Latino/Hispanic. The majority were females (81%) and spouses (51%). Main themes include, (a) Individual Level: Improvements in Caregiving Self-efficacy and Self-care Awareness, (b) Relational Level: Enhanced Communications and Relationships with Persons with Dementia, and (c) Community Level: Connection and Access to Community Resources and Support.

**Conclusion:**

Interview data show that the culturally and linguistically tailored program supported diverse caregivers by increasing self-care awareness, improving knowledge about dementia and dementia care, strengthening communication skills, and facilitating access to community resources. Strong rapport between CHWs and caregivers enhanced the effectiveness of the intervention. Future approaches can focus on supporting caregivers with especially limited resources.

## Introduction

By 2023, approximately 6.7 million Americans were living with dementia (Alzheimer’s Association, 2023). An estimation of 14 million older Americans are projected to have Alzheimer’s disease or related dementias by 2060 (Centers for Medicare & Medicaid Services, 2023). The progression of the disease eventually limits persons living with dementia from independently engaging in their everyday activities (Fieo et al., 2018). As a result, the need for caregivers is likely to grow in tandem with the projected increase of persons with dementia.

Many persons with dementia rely on unpaid informal caregivers, such as family and friends. An estimated 16 million Americans engage in combined 17 to 18 billion hours of informal caregiving to persons with dementia (Alzheimer’s Association, 2023). Daughters and spouses provide the bulk of care (Spillman et al., 2020). A third of the caregivers are over 65, and compared with caregivers of people without dementia, twice as likely to have significant financial, emotional, and physical difficulties (Alzheimer’s Association, 2023). Medicare claims data from 2021 estimate $321 billion associated with healthcare and long-term services for persons living with dementia and $271.6 billion for unpaid care from family and other informal caregivers (National Institute on Aging, 2022).

Caregiving is considered an independent risk factor for morbidity and mortality (Schulz & Beach, 1999). Continuous care for persons with dementia takes a significant toll on caregivers, resulting in the experience of chronic stress, depression, sleep disorders, poor health, low quality of life, and early mortality (Cheng et al., 2014). Caregivers’ poor health conditions raise the importance of understanding caregivers’ life outcomes and developing interventions that improve caregivers’ mental and physical health and reduce caregiving burden. Dementia caregiver interventions include diverse approaches such as psychoeducation, cognitive behavioral therapy, social support, and stress management (e.g., mindfulness) that take place in centers, on-line, or by telephone, with most applied to non-Hispanic White populations (Cheng et al., 2019). In comparison, dementia caregiver interventions among underserved minority family caregivers remain limited.

Racial and ethnic disparities exist in experiences with dementia care and caregiving due to sociocultural factors (Becerril et al., 2023; Idorenyin Imoh & Charity, 2023; Lee et al., 2022a; Parker et al., 2022; Quiroz et al., 2022; Vila-Castelar et al., 2022). In dementia studies, underserved caregivers from Latino/Hispanic and Asian communities encounter barriers that preclude quality healthcare for themselves and persons with dementia (Kenning et al., 2017; Lee et al., 2022a; Ta Park et al., 2019). Contributing factors include lack of culturally appropriate resources and linguistic barriers to accessing existing resources. Latino/Hispanic dementia caregivers struggle to find bilingual and bicultural support and information, leading to higher levels of distress and health disparities (Balbim et al., 2019; Caceres & Perez, 2018; Rote et al., 2019).

Asian Americans, particularly Vietnamese American and Korean American caregivers, encounter similar experiences (Kim et al., 2019; Nguyen et al., 2019). Little culturally tailored education on self-care and caring for persons with dementia is available from medical providers or other support groups due, in part, to underdiagnosis and underreporting of Alzheimer’s disease and related dementia (Kornblith et al., 2022; Mayeda et al., 2016). Consequently, this population is understudied in dementia research (Liu et al., 2020). Yet, as the fastest-growing racial group in the US, Asian Americans and their healthcare experiences warrant further attention (Pew Research Center, 2023). Asian American family caregivers for persons with dementia underutilize public health services for dementia care and tend to overlook treatment until situations become catastrophic or unmanageable due to language, time, and financial barriers (Kim et al., 2019).

Recent studies highlight cultural competence in healthcare and dementia care to help close racial/ethnic health disparities stemming from sociocultural barriers (Brijnath et al., 2022; Lee et al., 2022b; Rote et al., 2019; Waymouth et al., 2023). Cultural competence in healthcare systems include services and delivery that understand and consider the influence of culture on patient health beliefs and behaviors (Nair & Adetayo, 2019). This may include having culturally diverse service providers, making available linguistically accessible healthcare practitioners and staff, providing literature and materials that reflect an understanding of cultural histories, norms, and experiences, and creating culturally appropriate interventions and healthcare environments (Handtke et al., 2019; Nair & Adetayo, 2019).

This study uses interview data to examine caregivers’ experiences with a culturally and linguistically tailored home visit-based program. Understanding their experiences allows researchers to better grasp how the intervention benefits the caregivers across diverse racial/ethnic backgrounds and identify improvement areas.

## Methods

### Study design

The present study is part of a larger on-going randomized controlled trial parent study (Clinical Trials.gov, 2023). Family caregivers were randomly assigned in the intervention arm to receive a 3-month CHW-led home visit-based caregiving education. CHWs interviewed the caregivers at the end of the intervention to gather feedback on their participation experiences. Drawing from the interview data, thematic analysis identified intervention benefits. The institutional review board (IRB) of the authors’ affiliated university approved the study protocol prior to the start of the study.

### Participants and recruitment

We recruited family caregivers who care for their family members with dementia at home in California. The eligibility criteria for study participation included (a) adults aged 18 or older; (b) family caregivers, such as spouses/partners, adult children, and siblings, caring for a community-dwelling person with dementia; (c) caregivers reporting their ethnicity/race as Korean, Vietnamese, Latino/Hispanic, or non-Hispanic White; (d) caregiving experiences in guiding or assisting community-dwelling persons with dementia; and (f) at the time of study enrollment, no plan for long-term care placement for persons with dementia within 6 months. Family caregivers with any of the following conditions were ineligible for study participation: (a) cognitive impairment that precludes an individual from understanding the consent process and completing surveys (for those aged 65 or older as assessed by Mini-Cog© (Mini-Cog, 2023); (b) current active cancer (i.e., undergoing active treatment for cancer); or (c) need hospice care. We recruited participants by posting IRB-approved study flyers in local senior centers, adult day service centers, churches, non-profit organizations serving caregivers or individuals with Alzheimer’s, local medical offices serving ethnically diverse older adults, and federally qualified health centers. Word-of-mouth snowball methods were also used to reach family caregivers of persons with dementia.

### The culturally and linguistically appropriate CHW-led home-based intervention

Table 1 presents the 3-month program of the bilingual and bicultural CHW-led, home visit-based intervention. The in-home caregiver intervention comprises three components. The first entails demonstration and strengthening of stress reduction techniques such as mindfulness breathing. CHWs demonstrated mindful breathing which encourages participants to intentionally focus their energy on inhaling and exhaling for 2 min and sent reminders via messaging apps or text messages to practice the exercise. CHWs also helped caregivers identify stress triggers and stress by using multiple data points, including the caregivers’ subjective experiences and objective health metrics, rather than relying solely on the caregivers’ perception. The second component entails education on dementia caregiving skills to manage difficult behaviors including, (a) understanding symptoms and stages of dementia, (b) compassionate communication skills with persons with dementia, and (c) daily activities for persons with dementia to be completed alone or with the caregiver (e.g., puzzles, writing, reading, singing, taking a walk, etc.). Bicultural CHWs may suggest engaging in traditional games or singing along to popular cultural songs well-known to ethnic caregivers and persons with dementia. The third entails providing culturally appropriate resources for dementia care. Care resources include support groups, adult day centers, and other dementia-related social services (legal and financial matters), customized to accommodate the diverse cultural and instrumental needs of different ethnic and racial communities. For instance, CHWs visiting overburdened ethnic caregivers, would provide lists of culturally specific adult day centers that serve ethnic cuisine, employ staff with similar cultural backgrounds, and program cultural activities that are familiar to adults with dementia.

**Table 1.** The 3-month home-visit caregiving education intervention.

Week	Caregiving Education/Mindful Breathing and Compassionate Listening
Week 1	Mindful Breathing - Overview & practice session.
Home Visit 1	Compassionate Support/listening for caregiver.
	Education 1 - Understanding Alzheimer’s and related dementias & appropriate communication with persons with dementia.
Week 2	Mindful Breathing - Practice session & Compassionate Support/listening for caregiver.
Home Visit 2	Education 2 – Case-based practice to manage difficult behaviors of persons with dementia using video.
Week 3	Mindful Breathing - Practice session & Compassionate Support/listening for caregiver.
Home Visit 3	Education 3 – Care resources and activities to complete with persons with dementia.
Week 4	Mindful Breathing - Practice session & Compassionate Support/listening for caregiver.
Home Visit 4	Education 4 – Other issues (e.g., safety check, legal issue, end of life care preparation.)
Week 8	Mindful Breathing - Practice session & Compassionate Support/listening for caregiver.
Home Visit 5	Booster Home Visit 1 - Caregiver-driven topic discussion.
Week 12	Mindful breathing -practice session & Compassionate Support/listening for caregiver.
Home Visit 6	Booster Home Visit 2 - Caregiver-driven topic discussion.

Trained bilingual CHWs delivered the intervention in the participants’ homes every week for a month. Then the CHWs followed up with the participants monthly by text or phone calls until the 3-month mark (i.e., 12 weeks). Participants completed a face-to-face orientation at baseline and an exit interview at the end. Participants were allowed to call or text the CHWs to ask questions regarding caregiving or stress management during the 12-week intervention.

### Data collection and analyses

CHWs and trained research assistants collected demographic data at the baseline home visit using a survey. Caregiver participants reported their age, gender, relationship with persons with dementia, caregiver education, length of time in the U.S., medical comorbidity, and health insurance status (e.g., Medicaid, Medicare, or uninsured) along with care recipients’ age, gender, health insurance, type of dementia, and caregiver-self-reported level of dementia severity. Demographic data were analyzed using descriptive statistics in R (version 4.3.2) for Windows. Mean and standard deviation (SD) were calculated for continuous variables, and frequencies and percentage were calculated for categorical variables.

Qualitative data for this study drew from exit interviews with 57 caregivers. The semi-structured interview guide included questions about caregivers’ participation experiences, perceived behavior change, most useful caregiving skills, additional or unmet needs, and general feedback. Caregivers had opportunities to narrate their experiences or provide additional feedback they wanted to share. CHWs conducted the exit interviews at the end of the intervention. Having established a rapport with CHWs, the exit interviews offered a sense of closure for caregivers. The rapport also enabled caregivers to share their experiences more openly than otherwise with strangers as the interview questions were designed to safeguard the caregivers’ interests while gaining insights from their experiences.

While some interviews were completed in English, most were completed in the caregivers’ language of preference. Bilingual and bicultural CHWs transcribed and translated the interviews, and researchers familiar with the language reviewed the translation. In some cases, caregivers did not want the interviews recorded. In those cases, CHWs took detailed notes of the interviewees’ responses.

A multi-disciplinary team of researchers extracted and analyzed the interview data using a thematic analysis approach. Researchers from the health sciences considered the caregivers’ responses by bringing in experiences as healthcare providers to patients. Researchers with social sciences background contextualized the caregivers’ experiences by considering the caregivers’ social and cultural surroundings. The first author, two qualitative researchers, and a graduate student researcher independently reviewed the interviews, applying an inductive and deductive approach and noting patterns in the participants’ responses (Corbin & Strauss, 2015). The team systematically engaged in an iterative comparison process by meeting weekly to share impressions of the data, pinpoint similarities and differences in observations and underscore recurring themes. These regular comparisons and updates allowed the researchers to discern common experiences across different racial/ethnic groups as the data were collected.

After reading all the interviews and comparing the observations, the researchers developed a list of codes and defined each code. The team selected an interview to apply the developed list of codes. The researchers compiled the four coded documents, compared them, and refined the codes and their definitions. Four trained coders read the interviews line-by-line and applied the codes to all the interviews using ATLAS.ti. Each coder coded the documents independently. The coders met with the qualitative researcher after coding every five interviews to address discrepancies and ensure consistency in the coding approach until coding was complete. The first author, two qualitative researchers, and a graduate student researcher reviewed the coded documents, combining overlapping experiences into three overarching themes related to intervention benefits. Data saturation for the three themes was assessed when the sample size exceeded fifty caregivers, with at least ten participants from each of the four racial/ethnic groups. Intervention effects were identified at the individual level with the caregivers’ improvements in their own health management, at the relational level with the amelioration of strained relationships between caregivers and persons with dementia, and at the community level with increased access to community-based resources and social support networks.

## Results

Table 2 presents the demographic characteristics of caregivers and persons with dementia. Caregivers (*N* = 57) with a mean age of 63.5 (ranging 28–87) completed the 3-month intervention where 19 (33%) were Korean American, 16 (28%) Vietnamese American, 12 (21%) non-Hispanic White, and 10 (17.5%) Latino/Hispanic. The majority were female (81%), half (51%) were spouses, and nearly three quarters (74%) attained college or graduate education. Over half of the caregivers self-reported a comorbidity (53.6%). Caregivers who spoke Spanish, Vietnamese, or Korean self-reported moderate English proficiency (mean = 2.4 of 5, SD = 1.33). Among persons with dementia, 35% were female, mean age was 79.7 (ranging from 63 to 97), 42% had Medicaid, 47.4% had Alzheimer’s dementia with 38% in the early-stage and 41% in the middle-stage as reported by the caregiver.

**Table 2.** Characteristics of family caregivers and their family with dementia.

	Non-Hispanic Caucasian participants (*n* = 12)	Latino participants (*n* = 10)	Korean participants (*n* = 19)	Vietnamese participants (*n* = 16)	All (*N* = 57)
	Frequency (%) or Mean (SD)	Frequency (%) or Mean (SD)	Frequency (%) or Mean (SD)	Frequency (%) or Mean (SD)	Frequency (%) or Mean (SD)
Gender (female)	10 (83.3)	9 (90.0)	17 (89.5)	10 (62.5)	46 (80.7)
Age	69.67 (12.1)	51.60 (11.2)	68.5 (10.9)	60.87 (16.5)	63.6 (14.3)
Spouse	76.0 (7.6)	68 (0)	74.7 (6.5)	75.6 (6.8)	75.0 (6.7)
Adult Child	60.3 (7.8)	49.8 (10.2)	55.2 (3.4)	48.9 (12.6)	52.0 (10.0)
Caregiver relationship with person with dementia					
Spouse	8 (66.70)	1 (10.0)	13 (68.4)	7 (43.8)	29 (50.9)
Wife	6 of 8 (75.0)	1 of 1 (100)	10 of 13 (76.9)	3 of 7 (42.9)	20 of 29 (69.0)
Adult child	3 (25.0)	9 (90.0)	6 (31.6)	8 (50.0)	26 (45.6)
Daughter	3 of 3 (100)	7 of 9 (77.8)	6 of 6 (100)	6 of 8 (75.0)	22 of 27 (84.6)
Sibling	0 (0)	0 (0)	0 (0)	1 (12.5)	1 (1.7)
Education					
<=High School	0	6 (60.0)	2 (10.6)	7 (43.8)	15 (22.4)
>=College	12 (100)	4 (40.0)	17 (89.4)	9 (56.2)	52 (77.6)
Health insurance					
Yes	12 (100.0)	7 (70.0)	19 (100)	15 (100)	53 (94.6)
Medicaid	1 (8.3)	4 (40.0)	2 (10.5)	3 (18.8)	10 (17.5)
English proficiency^a^	Not applicable	2.20 (1.3)	3.05 (1.31)	2.88 (1.09)	2.44 (1.32)
Time living in US (years)	Not applicable	29.83 (10.9)	39.4 (13.9)	35.29 (10.1)	36.37 (12.3)
Comorbidity Health condition (Yes)	7 (58.3)	5 (50.0)	9 (47.4)	9 (60.0)	30 (53.6)
Persons with dementia (*N* = 57)					
Gender (female)	6 (50)	9 (90.0)	5 (26.3)	10 (62.5)	30 (52.6)
Age	82.08 (8.2)	79.40 (10.1)	80.3 (6.7)	77.3 (8.9)	79.7 (8.2)
Health insurance					
Medicaid only	1 (8.3)	2 (20.0)	2 (10.5)	1 (6.2)	6 (10.5)
Medicaid + Medicare	1 (8.3)	4 (40.0)	2 (10.5)	11 (68.8)	18 (31.6)
Medicare only	7 (58.3)	2 (20.0)	15 (78.9)	3 (18.8)	27 (47.4)
Uninsured	0	2 (20.0)	0	0	2 (3.5)
Types of dementia					
Alzheimer’s	7 (58.3)	3 (30.0)	11 (57.9)	6 (37.5)	27 (47.4)
Vascular	1 (8.3)	2 (20.0)	6 (31.6)	0	9 (15.8)
Lewy Body Dementia	0	1 (10.0)	0	0	1 (1.8)
Other	2 (16.7)	0 (0)	1 (5.3)	2 (12.5)	5 (8.8)
Do not know	2 (16.7)	4 (40.0)	1 (5.3)	8 (50.0)	15 (26.3)
Stage of dementia					
Early	2 (16.7)	3 (30.0)	12 (63.2)	4 (28.6)	21 (38.2)
Middle	5 (41.7)	2 (20.0)	7 (36.8)	3 (21.4)	17 (30.9)
Late	4 (33.3)	3 (30.0)	0	1 (7.1)	8 (14.5)
Do not know	1 (8.3)	2 (20.0)	0	6 (42.9)	9 (16.4)

^a^English proficiency measured with 5-Likert scale (4 = excellent, 0 = cannot speak English). Participants who were born in the U.S. were excluded in this mean calculation.

### Intervention exit interview

Caregivers reported benefits at the individual, relational, and community levels. At the individual level, caregivers described improvements in both caregiving capacity and self-care. At the relational level, caregivers experienced enhanced communications with persons with dementia and greater emotional connections with their family members with dementia. At the community level, CHWs connected the caregivers to community resources, providing instrumental support. More importantly, emotional support and affirmation of their caregiving experiences from the CHWs served as critical support stemming from outside the caregivers’ personal networks.

#### Individual level: Improvements in caregiving self-efficacy and self-care

After the 3-month intervention, caregivers recounted personal gains, including increased capacity to care for others and themselves. Commonly, spouses assumed the caregiving role by default. For others, such as adult children or siblings, family assigned greater responsibilities to unemployed members. A few participants left paid work for caregiving. As a result, many caregivers in this study had limited preparation and training in caring for persons with dementia in various stages. CHWs sought to fill the gap in caregiving knowledge and skills by providing individualized caregiver-centered educational resources and reviewing them with the caregivers to address stage-specific caregiving challenges. By the end of CHW’s home visits, caregivers expressed feeling empowered and more confident about meeting the needs of persons with dementia.

A non-Hispanic White husband already had knowledge about dementia prior to his wife’s diagnosis. Despite his knowledge, he had minimal caregiving experience and gradually developed caregiving strategies through trial and error over time. During the CHW visits, the husband learned new caregiving skills in a structured format. Specifically, the CHW helped him make connections between dementia symptoms and caregiving skills appropriate for those observed symptoms. The husband reflected,

I really did not have a good understanding before… now I have a better understanding as to what a caregiver is, what the roles are, what the conditions are, and how to cope with the situation. Once you know what’s causing them, then you can go from there.

79-year-old English-speaking husband

For less prepared caregivers, CHW visits offered steppingstones for learning about dementia, how to cope with care recipients’ present conditions, and how to prepare for future events.

In addition to developing knowledge and resources about caregiving, most caregivers gained information about self-care. Participants readily recognized the importance of monitoring the health conditions of persons with dementia. In contrast, most caregivers in this study failed to acknowledge the importance of self-care and monitoring one’s own health. Caregivers experienced mental and physical stress from the demands of caring for another, yet they rarely prioritized self-care. Caregivers also had few opportunities and strategies to manage their health. CHWs educated caregivers about how to identify stress triggers and symptoms and demonstrated stress reduction techniques, such as mindful breathing. Monitoring their own health in relation to caregiving helped caregivers improve awareness of their own health. CHWs also sent self-care reminders to caregivers through messaging apps or text messages. Some caregivers began making concerted efforts to respond to their own health needs. For example, a grandson caring for his grandfather with late-stage dementia experienced overwhelming stress. As his grandfather’s condition worsened, the caregiver’s responsibilities grew, and he practiced mindful breathing to regain control of himself in situations fraught with worries.

I practiced mindful breathing a couple of times when I felt overwhelmed… This technique gave me a minute to reset my mind. In that minute, I would stop thinking about the problem and stress that I was dealing with. It allowed me to collect my thoughts and think clearer. This technique helped me a lot in the stressful situations that I encountered.

34-year-old Vietnamese-speaking grandson

Caregivers additionally adopted more proactive approaches to self-care. One daughter recognized that her feelings of impatience and irritation indicate increased stress levels, even if she did not consciously perceive them as stress. Therefore, she practiced stress reduction techniques and began walking regularly, incorporating physical movement whenever she noticed shifts in her patience level. As discussed,

I frequently practiced the mindful breathing technique especially when I felt impatient during different situations… I also thought that the activity reminders motivated me to be more active and move more.

60-year-old Spanish-speaking daughter

Having completed caregiving classes and earned certificates from local organizations serving persons with dementia and their families, the Spanish-speaking daughter was an experienced caregiver. Therefore, she found educational materials from the CHW useful for refreshing her knowledge but less applicable because her mother already progressed to late-stage dementia. However, the CHW’s affirmation of her hard work and acknowledgment of her competence from family and friends gave her greater confidence and energy in her caregiving role.

Both newer and more experienced caregivers surprisingly often overlook self-care. Because many educational materials that caregivers accessed focused on care recipients’ conditions, caregiver questions also gravitated toward caring for person with dementia. As a result, caregivers placed care recipients’ health at the forefront and paid less attention to their own.

#### Relational level: Enhanced communication and relationships with person with dementia

CHW visits helped ameliorate strained relationships between caregivers and persons with dementia by strengthening connections and improving communications. Caregivers experienced frustration when persons with dementia exhibited symptoms or behaviors that caregivers did not understand. For newer caregivers, learning about the different stages of dementia and what to expect in each stage helped them better anticipate and accept behaviors of persons with dementia. CHWs also demonstrated compassionate listening and communication skills that caregivers could incorporate into their daily interactions with persons with dementia.

For example, a Spanish-speaking participant recently started caring for her mother-in-law. Initially, the mother-in-law’s frequent cravings for sweets and childlike expressions alarmed the caregiver. However, these behaviors have since become endearing. The daughter-in-law reflected, “knowing that certain behaviors are a normal part of dementia has helped me and my family not feel alarmed all the time.” A new understanding helped strengthen their relationship, both as family and as caregiver and person with dementia.

Interactions with persons with dementia became less stressful when caregivers recognized that unexpected or frustrating behaviors were symptoms of the condition. Caregivers described how they reoriented their emotions as they became more accepting of the behaviors associated with dementia and their caregiving roles. Caregivers responded to persons with dementia with greater patience and empathy. A Spanish-speaking caregiver had been caring for her husband for over ten years when he began displaying erratic behaviors. After his dementia diagnosis, the caregiver realized the behaviors as symptoms of dementia. Although her husband’s rapidly declining condition left the caregiver perplexed, education materials from the CHW helped her cope with the new caregiving challenges. The caregiver learned to refrain from correcting her husband when he made mistakes and adopted compassionate communication skills. She found a renewed connection and a strong desire to cherish their time together. She remarked, “I feel very close to him, and I always let him know that he’s my priority, and it makes me feel good. I’m more caring than I have been.”

One husband participated in the study because he wanted to be a better caregiver. He and his wife spent most of their time together, and the caregiver experienced numerous challenges. Traveling with his wife became an increased concern for the caregiver because he was worried about her safety. His wife often needed assistance using the toilet, but the caregiver had difficulties helping her. Although the caregiver enjoyed physical activities and playing golf, he had to reduce those activities. The CHW and the caregiver reviewed the progression of the wife’s condition and identified strategies to address the caregiver’s challenges, such as finding physical activities that the couple could enjoy together, hiring a paid caregiver, and practicing mindful breathing. The husband observed,

I think one of my shortcomings and weaknesses was the fact that I didn’t have a lot of patience and I was taking things more personally than I should have. When I was doing that, I could feel I was becoming aggravated and angry. Once I understood how and why that was happening, it became a lot easier to cope with it.

79-year-old English-speaking husband

In one father-daughter relationship, interactions were laden with confrontations. The daughter had been caring for her father, in his nineties, for over a decade. With severe cognitive impairment, he was prone to blaming his daughter for missed meals and complaining to other family members about it. During the 3-month period, the CHW and the caregiver worked on stress management and accepting her father’s complaints and forgetfulness as his symptoms. The daughter reflected,

Yes, I feel less stress when I am taking care of my dad right now. I would still get mad at my dad sometimes when he told others bad things about me. Before… I would fight back with him and correct his words in front of others. However, I learned that he might not know what he was talking about. I learned not to be mad at him by leaving him alone and distracting myself by working on house chores.

66-year-old Vietnamese-speaking daughter

Caregivers learned about compassion and respectful communication, with opportunities to cultivate these communication skills. Caregivers with limited English proficiency found educational materials tailored to their preferred language, detailing how to communicate and interact with persons with dementia in different stages of dementia, especially useful. Materials were culturally relevant and CHWs presented the information with cultural sensitivity. For example, a Korean-speaking wife caring for her husband with early-stage dementia found her husband’s excessive shopping habits and insistence on driving as troubling. Confronting her husband about the issues frequently resulted in anger and arguments. After CHW visits and learning about compassionate communication, the caregiver began to treat her husband with more respect and speak with a calmer voice. By the end of the intervention period, the husband willingly surrendered his car keys without resistance.

I think the most important skill I learned is how to speak with my husband. It was nice to learn it. I didn’t learn that elsewhere. I already know all the other things because I used to be a nurse, but communication skill is very important, and I realized that I need to know it well.

77-year-old Korean-speaking wife

Some caregivers had more complex communication issues because caregivers did not share the same language as persons with dementia. For example, communication between a daughter and her father with dementia was sparse because the daughter had limited Korean proficiency while her father experienced diminishing English abilities as dementia symptoms worsened. On a deeper level, the daughter and the father lacked a common cultural understanding which ripened the conditions for further misunderstandings. Bicultural CHWs helped bridge the gap by helping caregivers to recognize the cultural, non-cultural, and dementia-related factors shaping their caregiving circumstances. Few resources offer fully bilingual and bicultural support for caregivers and persons with dementia. Consequently, the daughter appreciated the educational materials and bicultural understandings that the CHW brought to the pair, which helped address the communication challenges exacerbated by culturally driven assumptions and misunderstandings. The daughter explained,

Written information in Korean was helpful for my father even if he didn’t read or understand [all] the contents. It has helped him to accept his limitations and not be defensive about his cognitive and memory decline. This allowed him to accept help more easily.

53-year-old English-speaking daughter

Communication between the caregiver and her father improved because both had access to the same information in their preferred languages. Meanwhile, the daughter recognized that her father struggled to accept his limitations and her assistance, as this altered the parent-child relationship and his patriarchal authority. The situation highlights the importance of bilingual and bicultural support when considering intergenerational differences in language acquisition and cultural understanding among families with immigration history.

#### Community-level: Access to community resources and broadening of support

CHWs enhanced caregiver connection and access to community-based resources as well as broadening the caregivers’ social support networks. CHWs helped bridge underserved minority caregivers’ unmet caregiving needs in two ways. One, CHWs improved caregivers’ access to community-level programs and services that effectively expanded the caregivers’ toolkit. Two, CHWs provided emotional support to the caregivers in ways that their personal networks could not.

CHWs played a critical role in making community-level resources more accessible to minority caregivers. A Vietnamese-speaking man caring for his wife applied for resources from community programs, but his application was denied. With a language barrier and lack of knowledge in application processes, he never appealed the decision. However, a CHW connected the caregiver with a Vietnamese community-serving organization. The organization assisted the husband with the application and helped him access the needed resources, which enhanced the husband’s caregiving capacity and prospects for both him and his wife. The caregiver expressed with gratitude, “I hope that this study later will become a service to make it more accessible to Vietnamese caregivers like me throughout the nation.” While all caregivers can potentially benefit from community resources, minority caregivers experience access barriers. Caregivers described limitations of the resources and support in their existing networks, and they valued specific ways that CHWs addressed those shortcomings.

In other cases, CHWs connected caregivers to support groups in the caregivers’ preferred language. One Korean-speaking daughter caring for her mother with mid-stage dementia experienced extreme levels of stress. Although she had family nearby, the caregiver felt unsupported and isolated. The CHW emphasized creating a support network for caregivers and the person with dementia. Because the caregiver had no time for travel, the CHW located Korean-speaking support groups that meet through teleconferencing. After attending the support group meetings, the caregiver remarked, “…knowing that there is a support group with a Korean community [was helpful]. It was so nice to have support meetings and participate in the dementia family support study through Zoom.”

In addition to addressing instrumental needs, CHWs provided culturally sensitive emotional support to the caregivers. As trained and certified bilingual paraprofessionals, CHWs visited the caregivers’ homes and developed trusted relationships. CHWs listened to daily caregiving challenges, and caregivers appreciated CHWs’ patience and understanding in their interactions. A Korean-speaking woman caring for her parent explained, “sympathy and understanding from the home visitor who has professional knowledge about dementia were so helpful to me. This is different from sympathy from family or friends. The home visitor validated my experiences.” Another caregiver described,

I like having the home visitor visit me. I get to share my concerns with the CHW. If I have any questions, the home visitor spent time talking and answering my concerns. It is hard to find a program that can send a person to visit the caregiver’s home and spend time talking to the caregiver. Therefore, I really appreciate that this study allows the home visitor to visit and talk to me.

66-year-old Vietnamese-speaking daughter

Caregivers underscored CHW support as helpful and validating, especially when compared to reactions from friends and family in their personal networks. Caregivers experienced disappointment when family and friends could not develop a deeper understanding of the caregiver’s situations, dismissed the caregiver’s worries, or displayed superficial sympathy. Repeated disappointments in existing relationships contributed to caregivers’ feelings of isolation. CHWs addressed the issue by helping caregivers unpack the isolating experiences of caregiving and unload emotional burden.

I also liked the personal touch of the home visitor and how she gave suggestions and listened and there was a back and forth sharing of reflections. I feel like that is the only time you really get to do that as no one in your family can sit with you and do that without bias.

67-year-old English-speaking wife

Other caregivers intentionally avoided talking to friends about the condition of their family members with dementia and the challenges of caregiving. Among these caregivers, common reasons include fear of rumors, stigma, and unwanted attention from friends. These caregivers viewed friends and neighbors as people to hide from rather than confide in. Caregivers’ experiences elucidate limitations in personal networks, especially when information in existing networks lacks relevant knowledge and understanding of dementia. For those caregivers, a lending ear from a trusted informed professional beyond the caregivers’ personal networks served as vital emotional support. CHWs connected caregivers to tangible community-based resources and, more importantly, helped the caregivers navigate isolating caregiving conditions that may have otherwise gone unnoticed.

Caregivers who were aware of community resources also benefited from CHWs. Many English-speaking participants and spousal caregivers already knew about caregiver support groups and local Alzheimer’s associations. Yet awareness of community groups and resources did not always lead to access and use of those resources. For instance, an English-speaking spousal caregiver knew about a local organization’s resources but had difficulty navigating its complex website to find the information that she needed. The CHW addressed the issue by focusing on one key tool that helps find local programs and events. The CHW demonstrated how to use the tool and spent time exploring it with the caregiver. The spousal caregiver recalled, “I really liked when you [the CHW] came out to give me some good ideas.” The CHW helped reduce the caregiver’s barriers to accessing community resources and improved the ease of navigating community-level information.

## Discussion

The overarching purpose of this study was to describe qualitative findings from an ongoing randomized controlled trial examining a culturally and linguistically appropriate home visit-based caregiving intervention for diverse caregivers led by CHWs. The intervention especially focused on caregivers from Latino/Hispanic and Asian American communities that require added cultural considerations (Kim et al., 2019; Meyer et al., 2020; Nguyen et al., 2020; Ta Park et al., 2019), as studies consistently report ethnic-racial variations in the psychosocial wellbeing of family caregivers (Liu et al., 2020; Moon et al., 2020). Caregivers in this study are also part of a growing population of older informal caregivers in the U.S. (Alzheimer’s Association, 2023), highlighting the importance of developing support systems that address the quality of life for both older caregivers and persons with dementia.

Analysis of the interview data allowed researchers to be reflexive about the intervention and its goals and improve implementation of the intervention. Findings show that rapport between CHWs and caregivers is vital for enhancing the effectiveness of the intervention, and the team made concerted efforts to minimize disruptions in the intervention by selecting and preparing CHWs who could commit for a longer term. The same CHW consistently visited the caregiver throughout the duration of the intervention, offering stability and dependability.

Experiences of stress and burden negatively affect caregivers’ quality of life and increase burnout, but person-centered interventions (Barbosa et al., 2015; Gassoumis et al., 2024) and interventions that consider the caregiver’s cultural and linguistic needs (Jaldin et al., 2023; Meyer et al., 2020) can improve the quality of life for family caregivers and persons with dementia. Findings from this study show that person-centered caregiver support, provided through culturally competent CHW-led home visits, mitigates adverse consequences and improves outcomes for family caregivers across three support levels: individual, relational, and community.

At the individual level, caregivers noted improvement in managing stress, care efficacy, and self-care. Caregivers in this study reported that education on stress management through mindful breathing was helpful as they adopted health management strategies. Caregivers acquired greater understanding of dementia and enhanced their caregiving efficacy by developing appropriate caregiving skills. Caregivers also emphasized the value of person-centered education on self-care and strategies for handling overwhelming situations, which they considered as among the most beneficial aspects of the CHW visits. This evidence is consistent with existing knowledge about the benefits of educational interventions aimed at developing dementia literacy, caregiving skills, and self-care for caregivers (Largent et al., 2021; Meyer et al., 2020; Sikora Kessler et al., 2021).

At the relational level, caregivers developed appropriate communication skills for interacting with persons with dementia. Applying communication skills based on compassion and empathy helped strengthen the relationship between the caregiver and person with dementia with healthier interactions. Family dynamics may worsen with the progression of symptoms in persons with dementia (Clemmensen et al., 2019) as caregivers in this study recounted. However, new communication skills can help mend relations between caregivers and persons with dementia relations as some caregivers expressed hope in repairing eroded relationships with person with dementia. While physical and mental health are important considerations, the quality of the relationship between caregivers and persons with dementia also deserves attention. Tailored caregiver emotional, social, and cultural support can improve care relationships (Bernstein et al., 2020). When caregivers develop better communication skills and a deeper understanding of the conditions affecting persons with dementia, caregivers and the relationships they nurture with persons with dementia both benefit.

At the community level, caregivers gained new connections or enhanced access to local resources. Connection to culturally relevant community resources can help reduce caregiver burden and stress (Abramsohn et al., 2022; Rote et al., 2017). Some ethnic caregivers with limited understanding of policies and procedural practices in this study were hesitant to apply for resources for which they qualified. The CHWs demystified the processes and served as liaisons, connecting caregivers to resources or removing access barriers, which increased caregivers’ use of community services. Importantly, caregivers received emotional and social support from the CHWs who were external to their personal networks. For many caregivers, regular home visits by CHWs who listened to their stories, affirmed their experiences, and provided informed advice alleviated feelings of frustration, burden, and isolation. The CHWs’ cultural understanding also produced an added benefit of building strong rapport and trust, which encouraged participation and completion of the intervention program.

Despite the benefits of bicultural CHW-led person-centered home visits, this study includes some limitations. First, the sample is not representative of the U.S. population and does not include the largest ethnic groups. Therefore, the generalizability of the findings needs to be considered for other ethnic/racial groups such as African Americans, Indigenous populations, and other Asian American and Pacific Islander populations. Second, the study only included caregivers whose family with dementia reside in the community. Those in skilled nursing facilities or other institutionalized settings (e.g., memory care facilities) were excluded. The intention was to improve the quality of care that family caregivers can provide at home, extending the duration of time that persons with dementia could continue living outside of a facility. Third, involving the CHWs in the exit interviews may situationally introduce social desirability bias where participants provide socially desirable or acceptable responses (Paulhus & Reid, 1991; Vassenden & Mangset, 2024). Participants may tend to give positive feedback due to the help they received from the CHWs or the relationship they built during the intervention program. However, trust between CHWs and participants can also encourage candid conversations as dementia care studies emphasize the importance of trust-building when working in underrepresented communities (Huggins et al., 2022; Koehn et al., 2022; Waymouth et al., 2023). Lastly, the model requires the availability of culturally competent bilingual CHWs to make home visits, which places considerable financial challenges and time demands. Further research needs to determine the effectiveness of exclusively virtual or hybrid models that integrate in-person and virtual intervention methods.

While caregivers share some common challenges, their experiences may vary geographically. Urban-rural health disparities and regional differences show that caregiving experiences, health outcomes, and service utilization vary spatially by race and ethnicity among caregivers providing dementia care (Kindratt et al., 2023; Yoshikawa et al., 2024). Because caregivers in the current study come from urban and suburban communities in California, caregivers have potentially easier and better access to community and ethnic resources than caregivers from other regions or rural communities in the US. For ethnic minority caregivers, geographic location may profoundly affect caregiving experiences. Caregivers embedded in larger immigrant and ethnic communities have opportunities to draw from ethnic community resources compared to caregivers in communities with scant ethnic resources. While our findings highlight the promise of having person-centered CHW home visits to caregivers, investigations in rural regions with limited community-based resources will be beneficial.

In conclusion, culturally and linguistically appropriate, bilingual CHW-led home-visit intervention was well-received by diverse dementia family caregivers. Participants reported the usefulness of education, community resources, and compassionate support delivered in their homes over 3 months. It is important that Alzheimer’s/Dementia-serving institutions recognize the need for cultural and linguistic tailoring, which does not stop at translation of documents. Family caregivers also demonstrated an uptake in use of dementia-related resources after engaging with the CHWs. Future studies could examine the impact of institutionalizing these services as part of public and/or private health insurance programs.
